# Screening Program for Asymptomatic Hepatitis C: Uptake and Detection in the Marche Region of Italy

**DOI:** 10.3390/jcm15083057

**Published:** 2026-04-16

**Authors:** Cecilia Acuti Martellucci, Sara Rosati, Matteo Fiore, Mosè Martellucci, Silvia Bizzarri, Margherita Morettini, Lucia Marinelli, Gianmarco Imperiali, Romina Fani, Francesca Brecciaroli, Silvia Scaramuzza, Jacqueline Orciani, Fabio Filippetti, Lamberto Manzoli

**Affiliations:** 1 Department of Medical and Surgical Sciences, University of Bologna, 40126 Bologna, Italy; matteo.fiore7@studio.unibo.it (M.F.); gianmarco.imperiali@studio.unibo.it (G.I.); lamberto.manzoli2@unibo.it (L.M.); 2 Department of Biomedical Sciences and Public Health, University of the Marche Region, 60020 Ancona, Italy; sara.rosati@sanita.marche.it (S.R.); silvia.bizzarri@sanita.marche.it (S.B.); 3 Department of Prevention, Local Health Unit of Ancona, 60035 Jesi, Italy; mose.martellucci@sanita.marche.it (M.M.); margherita.morettini@sanita.marche.it (M.M.); francesca.brecciaroli@sanita.marche.it (F.B.); 4 Department of Prevention, Local Health Unit of Macerata, 62100 Macerata, Italy; lucia.marinelli@sanita.marche.it; 5 Department of Prevention, Local Health Unit of Ascoli Piceno, 63100 Ascoli Piceno, Italy; romina.fani@sanita.marche.it; 6 Department of Prevention, Local Health Unit of Fermo, 63900 Fermo, Italy; silvia.scaramuzza@sanita.marche.it; 7 Department of Prevention, Local Health Unit of Pesaro Urbino, 61122 Pesaro, Italy; jacqueline.orciani@sanita.marche.it; 8 Healthcare Directorate, Regional Healthcare Agency of the Marche, 60125 Ancona, Italy; fabio.filippetti@regione.marche.it

**Keywords:** HCV, organized screening, early diagnosis, Italy, detection rate

## Abstract

**Objectives**: To report the performance of a screening program for undiagnosed HCV infections in the general population of one Italian region. **Methods**: The Marche region, central Italy, provided organized free HCV screening for the resident population born between 1969 and 1989, from July 2023 to December 2025. People with known liver disease or HCV treatment were excluded, and those eligible were invited by letter to access the blood draw. After a positive anti-HCV test, a reflex HCV-RNA test was performed on the same sample. The investigated outcomes were extension, uptake, HCV-RNA detection rate, and positive predictive value (PPV) of the anti-HCV test. Potential predictors of an infection diagnosis were investigated through multivariable analyses. **Results**: Over 30 months, 412,897 individuals were invited (93.6% extension), and 48,596 participated (11.8% uptake). Positive tests were 308 for anti-HCV (0.63%) and 42 for HCV-RNA (0.09% detection rate), with a relatively low PPV of the anti-HCV test (13.6%), which increased in males (15.2%) and individuals born in specific foreign countries (>20%). Multivariate analyses confirmed a higher risk of a positive HCV-RNA test for males (adjusted odds ratio—OR: 2.21; 95%CI 1.45–3.38) and those born in Moldova, Ukraine, and Pakistan (adjusted ORs between 10 and 15; *p* ≤ 0.001). **Conclusions**: The low detection rate was consistent with other Italian studies, suggesting that a combination of opportunistic recruitment and tailored pathways is required for high-risk groups in order to achieve the WHO HCV elimination targets. Future analyses should assess linkage-to-care and therapy compliance to evaluate the long-term cost–benefit ratio.

## 1. Introduction

In 2021, the Italian Government allocated over €70 million to develop free screening programs for the early diagnosis and treatment of Hepatitis C Virus (HCV) infections [1]. This was justified by the estimated 1.0% national prevalence of HCV chronic infection [2] and by the advent, in 2014, of direct-acting antiviral agents (DAAs), which drastically improved prognosis through a sustained virological response [3,4]. This advance in treatment reduced disease complications and led to economic benefits for health systems [5,6], prompting the implementation of HCV screening not only for high-risk groups but also for the general population, which is also likely cost-effective, depending on the prevalence of infection [7,8]. Several programs have been initiated worldwide in the last decade, either directed at patients in hospitals and emergency departments or within addiction clinics or in asymptomatic cohorts of individuals at average risk, such as the “baby boomers” born between 1945 and 1965 [7,9]. Since 2021, several Italian regions have launched screening programs for the general population born between 1969 and 1989; however, only two assessments of their performance are available to date, from Lombardy and Emilia-Romagna, together with one ad interim study from the Pesaro-Urbino Province [10,11]. In July 2023, the Marche Region, central Italy, started a population-wide screening campaign to identify adults with chronic HCV infection and refer them for antiviral treatment [12]. This work reports on the results of the 30-month screening program in terms of extension, uptake, detection rates, and predictors of an HCV infection diagnosis.

## 2. Materials and Methods

### 2.1. Study Design and Participants

We carried out an observational, descriptive study to assess the performance of the HCV screening program in the five Local Health Units (LHUs) of the Marche Region, from its beginning in July 2023 up to its completion in December 2025. Instead, the high-risk groups, i.e., prison inmates and people who inject drugs (PWIDs), were targeted by previous, separate interventions [13,14], and the current screening was directed towards the general population. Inclusion criteria were (A) being domiciled in the Marche Region, which is verified through registration with a general practitioner of the regional healthcare service, and (B) belonging to the birth cohorts from 1969 to 1989. Exclusion criteria were (A) being in treatment with anti-HCV drugs, (B) having a co-pay exemption for active chronic hepatitis, and (C) having a co-pay exemption for liver cirrhosis or biliary cholangitis.

### 2.2. Inclusion in the Screening Program

The eligible subjects were invited through a standardized letter, which contained information on the purpose and procedures of the screening and only varied in the instructions for participation. The eligible population, type of screening tests, and final referral to specialist care were uniform across all LHUs. However, the recruitment process varied: depending on the LHUS, the individuals could request the test (A) directly, just showing up to the laboratory; (B) directly, after an online or phone reservation; or (C) after a verification of the eligibility criteria by a physician.

### 2.3. HCV Testing Procedures

A single venous blood sample was taken, preserved, and tested for HCV in the Regional Health Service laboratories following the acquisition of signed informed consent. The examination was free, and no prescription was needed from the general practitioner.

The sample was then processed in two steps: the first-level test was performed to identify anti-HCV IgG antibodies through chemiluminescent microparticle immunoassay (CMIA) ALINITY Anti-HCV assay (Abbott Diagnostics, Lake Forest, IL, USA) on the ALINITY ci-series, or through indirect chemiluminescent immunoassay (CLIA) LIAISON XL Murex HCV Ab assay (DiaSorin S.p.A., Saluggia, Italy) on the LIAISON XL analyzer. If the first test was positive, a second (reflex) test was performed, searching for HCV-RNA through quantitative real-time polymerase chain reaction (RT-PCR) assays using either the BIORAD CFX96 Real Time System (Bio-Rad Laboratories, Hercules, CA, USA) or the Cobas 6800 System (Roche Diagnostics Ltd., Burgess Hill, UK). In each LHU, two to three laboratories routinely performed the anti-HCV test, while only one central laboratory performed the HCV-RNA test. The subjects who were positive for the HCV-RNA test, therefore diagnosed with an active infection, were referred to the gastroenterology hub of each LHU. The test findings were transmitted to LHU data centers via an online interface. In compliance with European General Data Protection Regulation (GDPR) policies [14], anonymized data were then shared with the Marche Region Health Department, which was explicitly requested to perform an evaluation of the program by the Italian Ministry of Health.

### 2.4. Outcomes

The study outcomes are the following, based on the methodology by Imperiali et al. [11]:

1. Screening extension: the percentage of people invited for the screening out of all those eligible;
2. Anti-HCV screening uptake: the percentage of people who took the anti-HCV test out of all those invited;
3. Proportion of tests positive at the anti-HCV test;
4. HCV infection detection rate: the percentage of people who were positive at the confirmatory HCV-RNA test out of those who took the anti-HCV test;
5. Positive predictive value (PPV) of the anti-HCV screening test: the percentage of people who were positive at the confirmatory HCV-RNA test out of those who were positive at the anti-HCV test.

### 2.5. Statistical Analysis

The collected data consisted of gender, age, country of birth, and results of the anti-HCV and HCV-RNA tests performed between 1 July 2023 and 31 December 2025. Descriptive statistics were employed to assess the sample’s key features and outcomes, and then multivariable random-effect logistic regression models were fit to investigate potential independent predictors of resulting positive anti-HCV and HCV-RNA tests, using the local health units as cluster variables. The model predicting anti-HCV-positive tests was adjusted a priori for all the available variables, alternating country of birth (Italy vs. abroad) to area of birth (specific areas of Italy and other countries). Instead, in order to maintain a covariate-to-failure ratio within 1:10, the model predicting positive HCV-RNA tests was built using stepwise selection, keeping only the covariates with *p* < 0.05 in the final model. All analyses were performed using STATA 15.1 (Stata Corp., College Station, TX, USA, 2017).

## 3. Results

Overall, 441,330 residents of the Marche Region were eligible for the HCV screening program from July 2023 to December 2025 (Table 1). Of them, 412,897 were invited to participate (extension: 93.6%), and 48,596 subjects were screened, for an uptake of 11.8%, with marked variability across LHUs.

Specifically, the LHUs with direct access obtained on average 9.5% (LHUs 1, 4, and 5—without reservation, but also LHU 3—with reservation), whereas LHU 2, where physicians verified eligibility before testing, obtained a significantly higher uptake (15.4%; *p* < 0.001).

The individuals testing positive at the anti-HCV test were 308 (0.63%). Of them, 42 were also HCV-RNA positive, corresponding to a detection rate of 0.09%, which did not differ significantly by LHU. The positive predictive value of the anti-HCV test for a diagnosis of chronic infection was 13.6%, and it varied from 6.2% to 19.6% across the five LHUs.

The mean age of those screened was 47.0 ± 6.0 years, 61.0% were female, and 81.9% were born in Italy (Table 2). The rate of those positive at the anti-HCV test was higher among males versus females (0.83% vs. 0.51%; *p* < 0.001); among those aged 51–57 years, as compared with those aged 34–40 years (0.76% vs. 0.48%; *p* < 0.001); and generally among the persons who were born abroad versus those born in Italy (1.10% vs. 0.53%; *p* < 0.001). At multivariable analyses (Table 3), significant independent predictors of being anti-HCV positive were male sex (OR 1.73, 95% CI: 1.27–2.35) and age above 50 years (OR 1.80; 1.42–2.28). Also, the individuals who were born in Moldova, Ukraine, and Pakistan showed approximately eight- to twelve-fold greater odds of anti-HCV positivity compared to those born in Italy.

Concerning HCV chronic infections (positive HCV-RNA test), detection rates were significantly higher among males than females (1.3‰ vs. 0.6‰; *p* = 0.005) and among the subjects who were born abroad (1.7‰ vs. 0.7‰ among those born in Italy; *p* < 0.001). Multivariable analyses confirmed a significant positive association with male gender (OR = 2.21; 1.45–3.38) and country of birth: similar to the anti-HCV test, individuals from Moldova, Ukraine, and Pakistan exhibited a ten- to sixteen-fold increase in the odds of HCV-RNA positivity (Table 3).

Predictably, the higher likelihood of a chronic infection among the individuals from higher-risk geographic areas determined an improved performance of the HCV screening in these groups: the positive predictive value of anti-HCV tests resulted in over or equal to 20% among persons from Romania, India, Moldova, Ukraine, Pakistan, and also Italy’s islands (Table 2).

## 4. Discussion

The Marche Region carried out one of the first Italian population-wide organized HCV screening programs, targeting the 1969–89 birth cohorts. In the 30-month program, we found a relatively low: (a) prevalence of HCV infection in the general population (detection rate: 0.09%); (b) screening uptake (11.8%), although it was significantly higher when the physicians verified eligibility before testing; and (c) positive predictive value of the anti-HCV test, although it was higher among the males and those born in specific foreign countries.

The detection rate observed in this study was very similar to the one reported by two HCV screening programs from other Italian regions: Emilia-Romagna, which used both organized and opportunistic invitations, reporting a detection rate of 0.12% [11], and Lombardy, which adopted an exclusively opportunistic approach, with a detection rate of 0.10% [15]. Taken together, these results suggest that the Italian HCV chronic infection prevalence may be closer to the Western European countries (3‰) and substantially lower than the 1% calculated by the Polaris Observatory [2]. Should this data be confirmed by further regional screening programs, the number of individuals that should be tested (number needed to screen—NNS) [16] to identify one undiagnosed HCV infection in the 1969–1989 cohorts would shift from the originally postulated 100 (1% prevalence) [2] up to 833–1111 (0.12% to 0.09% prevalence).

Concerning screening uptake, the Marche Region reached almost 12%, which is higher than the 4% uptake of the Lombardy Region, but much lower than the 37% of the Emilia-Romagna Region [11,15]. Unlike the present study, where only letter invitations were sent, Emilia-Romagna combined scheduled invitations with opportunistic testing at routine blood draws and supported participation through SMS, electronic health record reminders, and dedicated media campaigns [11]. In contrast, the Lombardy program limited recruitment to hospital wards, blood collection centers, and day hospital services [1]. On one hand, the lower participation elicited by a strictly opportunistic approach is confirmed by a report from West Virginia, where opportunistic screening obtained a 7% uptake [17]. On the other hand, the much higher uptake of the Emilia-Romagna, combined with the differences observed between Marche’s LHUs, may suggest a substantial positive effect due to advertising the screening through multiple strategies and to having physicians assess the participants at the blood draw. Two further studies support these findings, one from Lithuania and one from Maryland, which involved primary care clinics in the recruitment process, and reached 44% uptake (both studies) [18,19]. Indeed, provider education was consistently shown to improve participation in HCV screening [20], and a previous project conducted in the Marche Region also observed an improved uptake of fecal immunochemical testing when primary care physicians were actively involved [21].

In the present screening, one out of seven subjects with positive anti-HCV IgG tests was positive for the HCV-RNA, and the overall PPV was also in line with or slightly lower than that reported in previous Italian studies (15–36%) [1,11,22,23,24,25], probably due to the exclusion of subjects with known liver disease, which was unique to the Marche screening program, and may have lowered the prevalence of chronic infection in the screened population.

Notably, the higher odds of chronic infection and, consequently, the higher PPV reported for some subgroups (e.g., males, specific age groups, and those born abroad) might reflect different, for instance, repeated exposure patterns. The higher seroprevalence in males is generally attributed to a more widespread exposure to injection drugs and a lower likelihood of spontaneously clearing the virus [26,27,28]. Concerning the age groups, the rate of positive HCV-IgG tests increased with age, in agreement with virtually all the published figures [29,30,31,32,33]. Indeed, HCV testing became available in 1990 and, together with improvements in hygiene and socioeconomic conditions, contributed to limiting transmission, leading to a cohort effect in the national seroprevalence [34,35,36,37]. However, in the present study, the detection of HCV-RNA did not increase with age, an irregular trend that contrasts with the results of the Lombardy program [15], but is consistent with two recent prevalence studies [32,33]. Specific trends in transmission routes may be responsible for this phenomenon: the 1990s saw a rapid surge in the use of injection drugs [38,39], which was not coupled with a growing risk awareness, as restrictions on blood donor eligibility were enforced only in 2002 [40]. However, most importantly, the fluctuating infection prevalence across age groups is likely attributable to the very low number of HCV-RNA-positive individuals. Indeed, age was not included in the multivariable regression models predicting infection, as it showed no statistically significant association with being HCV-RNA positive.

Another predictor of an HCV chronic infection was the area of birth, with an unexpectedly high PPV among people born in Italy’s islands. This may be a random finding due to the very small sub-sample (1.1% of the study population) and is not supported by screening results from Sardinia, which has no data published yet, and Sicily, where the detection rate is 0.8%, according to preliminary data published by the national HCV Screening Dashboard (5.4% uptake) [41].

Conversely, the markedly higher PPVs recorded among people born outside of Italy were expected. Indeed, in recent decades, high-income countries have virtually eliminated iatrogenic infections from transfusions and medical equipment [29,42,43], whereas some low- and middle-income countries still struggle with these hazards [44]. For instance, in Pakistan, the mean HCV-RNA prevalence was estimated at 6.2% by one meta-analysis [45]. Also, several papers confirm the findings of a high prevalence (2.9%) of chronic infection in Eastern Europe and the former Soviet Union, where the combination of iatrogenic and drug-abuse exposures led to widespread transmission [2,46,47,48]. The higher prevalence observed in foreign-born residents of the Marche Region places this group in a similar risk category as the individuals who inject drugs and prison inmates [11,13], suggesting that they could be targeted by tailored micro-elimination strategies.

The recommendation to offer free HBV and HCV testing to all newly arrived migrants is not new; however, it presents two inherent setbacks [49,50]. The first counterargument is that risk differs by country of origin: for instance, migrants from African nations were shown to have a relatively low prevalence of chronic HCV infection (0.7%) upon arrival in Italy [51], and to predominantly get infected after residing in Italy for at least one year [52,53]. This was confirmed by the very low PPV observed in the present study, which included data on almost 1400 African nationals. The second concern lies with missing the unapparent infections of the larger local population. In the Marche Region, foreigners represent around 9% of the total [54], and they accounted for 36% of the infections, meaning that if they had been the exclusive target of the screening, only 15 infections would have been detected. At the same time, evidence indicates that although immigrants generally settle in clusters [55], they share transmission pathways with the local population, not only when engaging in risky behaviors such as injection-drug use and sex work [56,57,58,59], but also, more commonly, through caregiving work, which sees many immigrant women living in close contact with the elderly [60]. As a consequence, it is possible that targeting primarily foreigners from high-risk countries could reduce costs, and result not only in avoided morbidity and mortality for those chronically infected, but also in reduced transmission, mainly in foreigners, but also in the autochthonous population [61,62].

Among the strengths of the study are the large sample (the screening reached over 410,000 eligible residents of one Italian region, and tested almost 50,000 subjects), the exclusion of the individuals with a prior diagnosis of liver disease, which made it possible to estimate the rate of chronic infection only among asymptomatic individuals, and the fact that the laboratories adopted the reflex testing protocol, processing single blood samples according to a validated two-step technology. Nevertheless, there are limits to the study that must be taken into account. First, the validity of the anti-HCV test could not be evaluated, since those who tested negative did not take the HCV-RNA test. Second, no clinical data were available; therefore, only the demographic characteristics could be analyzed as potential predictors of HCV infection. Third, data on the overall invited population and on the subsequent linkage-to-care (e.g., potential predictors of uptake, visit adherence, and drug therapy uptake) were not collected, limiting the scope of the performance evaluation. Indeed, the available evidence suggests that the rate of successful linkage-to-care varies greatly across different settings and screening strategies, and may thus heavily influence the final impact of screening programs [9,20,63].

## 5. Conclusions

In conclusion, the HCV screening program of the Marche Region, targeting the general population born between 1969 and 1989, yielded a relatively low participation and a chronic HCV infection detection rate (positive HCV-RNA tests) of 0.1% instead of the expected 1%. The positive predictive value was greater among men and those born in specific foreign countries. In order to achieve the HCV elimination targets recommended by the WHO, provided that appropriate resources are available, policymakers should consider including physician evaluations when performing the blood draw, together with opportunistic recruitment, as tools to improve participation, and also devising tailored pathways for the identified high-risk groups, to improve detection rates. Future analyses should verify screening results from multiple Italian regions, in order to update the national prevalence estimates, and possibly assess linkage-to-care and therapy compliance data, to evaluate the long-term cost–benefit ratio.

## Figures and Tables

**Table 1:** HCV screening invitations, extension, uptake, positive test rates, and positive predictive value, by local health unit (LHU) of the Marche region.

Performance Parameters	Overall	LHU 1	LHU 2	LHU 3	LHU 4	LHU 5
Eligible subjects ^A^, N	441,330	101,288	155,084	81,017	48,152	55,789
Invited subjects ^B^, N	412,897	95,926	145,205	72,790	45,337	53,639
Extension, %	93.6	94.7	93.6	89.8	94.2	96.1
Participating subjects ^C^, N	48,596	10,582	22,352	6931	3250	5481
Uptake, %	11.8	11.0	15.4	9.5	7.2	10.2
Positive anti-HCV test, N	308	87	81	46	29	65
Positive at anti-HCV, %	0.63	0.82	0.36	0.66	0.89	1.19
Positive HCV-RNA test, N	42	11	14	9	4	4
HCV infection detection rate, %	0.09	0.10	0.06	0.13	0.12	0.07
Positive predictive value ^D^, %	13.6	12.6	17.3	19.6	13.8	6.2

^A^ Eligible residents (on 1 January 2024) born between 1969 and 1989, according to the Regional Vital Registration Statistics. ^B^ Eligible residents invited between 7 July 2023 and 31 December 2025, excluding the invitations that did not reach the recipient. ^C^ Invited residents tested between 1 August 2023 and 20 January 2026. ^D^ Rate of subjects positive at the HCV-RNA test out of those positive at the anti-HCV test.

**Table 2:** Characteristics of the participants to the screening, overall and by results of anti-HCV and HCV-RNA tests.

Characteristics	Overall, % * (N = 48,596)	Anti-HCV IgG +, % * (N = 308)	*p* ^†^	HCV-RNA +, % * (N = 42)	*p* ^†^	Positive Predictive Value ^‡^, %
Gender						
- Female	61.0	0.51	<0.001	0.06	0.005	12.0
- Male	39.0	0.83		0.13		15.2
Age in years ^A^, mean (SD)	47.0 (6.0)	48.1 (5.7)	0.001	46.9 (6.0)	0.15	-
Age categories						
- 34 to 40	18.5	0.48	0.022	0.09	0.031	18.6
- 41 to 45	20.2	0.52		0.10		19.6
- 46 to 50	26.2	0.67		0.06		9.4
- 51 to 57	35.1	0.76		0.09		12.4
Country of birth						
- Italy	81.9	0.53	<0.001	0.07	<0.001	12.8
- Abroad	18.1	1.10		0.17		15.5
Area or country of birth						
- Marche Region and central Italy	70.2	0.52	<0.001	0.06	<0.001	11.9
- North Italy	3.6	0.75		0.12		15.4
- Southern Italy	7.0	0.58		0.06		10.0
- Islands Italy	1.1	0.36		0.36		100
- Romania	2.4	0.59		0.17		28.6
- All African countries	2.8	1.09		0.07		6.7
- India	0.6	1.37		0.68		50.0
- Moldova	1.0	3.18		0.64		20.0
- Ukraine	0.6	3.41		0.68		20.0
- Pakistan	0.5	5.53		1.28		23.1
- Other countries	13.0	0.76		0.05		6.1

* Percentage unless otherwise specified. ^†^ Chi-squared test for categorical variables, Kruskal–Wallis test for continuous ones. ^‡^ Rate of subjects positive at the HCV-RNA test out of those positive at the anti-HCV test. ^A^ Age on 31 December of the year when the test was performed.

**Table 3:** Multivariable logistic regression, using LHUs as the cluster variable, predicting the results of anti-HCV and HCV-RNA tests (N = 48,596).

Characteristics	Anti-HCV IgG +			HCV-RNA +		
	OR	(95% CI)	*p* *	OR	(95% CI)	*p* *
Gender						
- Female	1.00	(ref. cat.)	-	1.00	(ref. cat.)	-
- Male	1.73	(1.27–2.35)	0.001	2.21	(1.45–3.38)	<0.001
Age categories ^A^						
- 34 to 40	1.00	(ref. cat.)	-	-	-	-
- 41 to 45	1.12	(0.80–1.58)	0.5	-	-	-
- 46 to 50	1.53	(1.08–2.19)	0.018	-	-	-
- 51 to 57	1.80	(1.42–2.28)	<0.001	-	-	-
Country of birth						
- Italy	1.00	(ref. cat.)	-	1.00	(ref. cat.)	-
- Abroad	2.23	(1.61–3.11)	<0.001	2.66	(2.25–3.14)	<0.001
Area of birth ^B^						
- Marche Region and central Italy	1.00	(ref. cat.)	-	-	-	-
- North Italy	1.42	(1.10–1.82)	0.006	-	-	-
- Southern Italy	1.09	(0.62–1.92)	0.8	-	-	-
- Islands Italy	0.71	(0.19–2.69)	0.6	-	-	-
- Romania	1.29	(0.60–2.80)	0.5	-	-	-
- All African countries	2.19	(1.06–4.54)	0.035	-	-	-
- India	2.74	(1.69–4.45)	<0.001	-	-	-
- Moldova	7.57	(3.56–16.1)	<0.001	-	-	-
- Ukraine	8.03	(5.37–12.0)	<0.001	-	-	-
- Pakistan	11.9	(8.69–16.3)	<0.001	-	-	-
- Other countries	1.36	(1.04–1.94)	0.024	-	-	-
Area of birth ^C^						
- All other countries	-	-	-	1.00	(ref. cat.)	-
- Moldova	-	-	-	10.5	(2.90–37.7)	<0.001
- Ukraine	-	-	-	11.9	(2.94–48.1)	0.001
- Pakistan	-	-	-	15.6	(4.63–52.6)	<0.001

OR = adjusted odds ratio. CI = Confidence Interval. * Wald test from the logistic regression. ^A^ Age on 31 December of the year when the test was performed. ^B^ ‘Country of birth’ and ‘Area of birth’ were included in two separate models predicting positive anti-HCV tests. ^C^ Specific countries were included in the model predicting positive HCV-RNA tests using stepwise selection, to avoid overfitting, in addition to ‘Country of birth’.

## Data Availability

The data presented in this study are available on request from the corresponding author, as approval by the Marche Region Healthcare Directorate is needed for data sharing.

## Abbreviations

The following abbreviations are used in this manuscript:

HCV	Hepatitis C Virus
LHU	Local Health Unit
PPV	Positive predictive value
NNS	Number needed to screen
WHO	World Health Organization
